# Association of ECG parameters with late gadolinium enhancement and outcome in patients with clinical suspicion of acute or subacute myocarditis referred for CMR imaging

**DOI:** 10.1371/journal.pone.0227134

**Published:** 2020-01-10

**Authors:** Kady Fischer, Maximilian Marggraf, Anselm W. Stark, Kyoichi Kaneko, Ayaz Aghayev, Dominik P. Guensch, Adrian T. Huber, Michael Steigner, Ron Blankstein, Tobias Reichlin, Stephan Windecker, Raymond Y. Kwong, Christoph Gräni

**Affiliations:** 1 Department of Cardiology, Inselspital, Bern University Hospital, University of Bern, Bern, Switzerland; 2 Department of Anaesthesiology and Pain Medicine, Inselspital, Bern University Hospital, University of Bern, Bern, Switzerland; 3 Non-invasive Cardiovascular Imaging, Cardiovascular Division, Department of Medicine, Brigham and Women’s Hospital, Harvard Medical School, Boston, MA, United States of America; 4 Non-invasive Cardiovascular Imaging, Department of Radiology, Brigham and Women’s Hospital, Harvard Medical School, Boston, MA, United States of America; 5 Department of Diagnostic, Interventional and Paediatric Radiology, Inselspital, Bern University Hospital, University of Bern, Bern, Switzerland; Ospedale del Cuore G Pasquinucci Fondazione Toscana Gabriele Monasterio di Massa, ITALY

## Abstract

**Background:**

Risk stratification of myocarditis is challenging due to variable clinical presentations. Cardiovascular magnetic resonance (CMR) is the primary non-invasive imaging modality to investigate myocarditis while electrocardiograms (ECG) are routinely included in the clinical work-up. The association of ECG parameters with CMR tissue characterisation and their prognostic value were investigated in patients with clinically suspected myocarditis.

**Methods and results:**

Consecutive patients with suspected myocarditis who underwent CMR and ECG were analysed. Major adverse cardiovascular event (MACE) included all-cause death, hospitalisation for heart failure, heart transplantation, documented sustained ventricular arrhythmia, or recurrent myocarditis. A total of 587 patients were followed for a median of 3.9 years. A wide QRS-T angle, low voltage and fragmented QRS were significantly associated with late gadolinium enhancement. Further, a wide QRS-T angle, low voltage and prolonged QTc duration were associated with MACE in the univariable analysis. In a multivariable model, late gadolinium enhancement (HR: 1.90, 95%CI: 1.17–3.10; p = 0.010) and the ECG parameters of a low QRS voltage (HR: 1.86, 95%CI: 1.01–3.42; p = 0.046) and QRS-T-angle (HR: 1.01, 95%CI: 1.00–1.01; p = 0.029) remained independently associated with outcome. The cumulative incidence of MACE was incrementally higher when findings of both CMR and ECG were abnormal (p<0.001).

**Conclusion:**

In patients with clinically suspected myocarditis, abnormal ECG parameters are associated with abnormal tissue characteristics detected by CMR. Further, ECG and CMR findings have independent prognostic implications for morbidity and mortality. Integrating both exams into clinical decision-making may play a role in risk stratification in this heterogeneous patient population.

## Introduction

Myocarditis is an inflammatory disease of the heart muscle, associated with acute and chronic heart failure and sudden cardiac death[1,2]. Due to the heterogeneity of the clinical manifestations, establishing the diagnosis is challenging[3]. CMR has the ability to characterise the tissue for necrosis, fibrosis and oedema, known features of myocarditis, using late gadolinium enhancement (LGE) and T2 weighted (T2w) imaging[3,4]. As a part of the clinical assessment electrocardiogram (ECG) is routinely performed and provides information on burden of cardiomyopathy and electrical instabilities. Historically ECG changes are nonspecific and not independently useful in diagnosing and prognosing myocarditis since there was no non-invasive gold standard. With the advent of CMR, macroscopic focus and the extent of myocardial scar can be readily detected and sized, so it creates an opportunity for us to better understand how to extract important information from the ECG that is clinically useful. Unspecific ECG abnormalities are described in myocarditis patients including QRS-T angle deviations, T-wave inversion, diffuse ST-segment changes or PR depression[5,6]. Further, conduction disorders such as fragmented QRS complexes (fQRS) have established associations with myocardial scarring, LGE, in patients with coronary artery disease, dilated cardiomyopathy and hypertrophic cardiomyopathy with good specificity and positive predictive value[7,8]. ECG parameters such as fQRS and QRS-T angles may serve as a simple, low-cost, non-invasive and readily available tool in the diagnostic and prognostic approach. However, their association with CMR findings and with outcome in patients with suspected myocarditis is not fully established.

In the current study, we sought to analyse the association of ECG parameters with imaging parameters derived from CMR in patients with clinically suspected myocarditis. Furthermore, in this cohort we analysed the prognostic value of ECG and CMR parameters in a combined statistical model.

## Methods

### Patient population

Data was collected from 2002–2015 in a single-centre study (NCT03470571). The detailed study design and primary results, of this retrospective study have been published previously[9,10]. Consecutive patients who were referred to a contrast-enhanced CMR by their treating physician with the primary clinical suspicion of myocarditis were considered if LGE and ECG information was available and clinical signs were documented. These consisted of either the acute presentation of chest pain syndromes and symptoms within the previous two weeks, or sub-acute (two to four weeks) signs of dyspnoea, left-ventricular dysfunction, abnormal ECG’s including ventricular arrhythmias and syncopal spells. Final inclusion for this analysis was based on if patients also met diagnostic criteria proposed by a position statement of the European Society of Cardiology Working Group on Myocardial and Pericardial Diseases, supported by an American Heart Association scientific statement[11,12]. To meet these diagnostic criteria, patients had to fulfil at least one category composed of ECG abnormalities, elevated troponins, cardiac functional and structural abnormalities, or positive tissue characterization of LGE in typical myocarditis patterns and or oedema. All patients with any evidence of coronary artery disease were excluded from the analysis. Exclusion criteria also included any prior clinical evidence or CMR characteristics for severe valve disease stress cardiomyopathy constrictive pericarditis, Loeffler endocarditis, left ventricular non-compaction, cardiac tumour, pulmonary embolism, along with infiltrative cardiomyopathies such as hypertrophic cardiomyopathy, arrhythmogenic right ventricular cardiomyopathy, cardiac sarcoidosis and cardiac amyloidosis. Laboratory blood markers of creatinine kinase, c-reactive protein, troponins I and T, white blood cell count, and N-terminal pro-brain natriuretic peptide (NTproBNP) were performed at the discretion of the clinician and were not systematically collected for the entire population. Similarly, endomyocardial biopsies results were available for 56 patients.

### ECG acquisition and analysis

Resting 12-lead ECG recordings were recorded with standardised procedures at the time of the CMR exam and assessed blinded to CMR findings and patient identity. ECG parameters of historical interests in detecting or prognosticating patients with myocarditis were included. We included low-QRS voltage, defined by ≤5 mm in limb leads or ≤10 mm in all precordial leads[13], fragmented QRS, defined as QRS complexes with the presence of an additional R wave (rSr ′, RSR′) or notching in either the nadir of the R wave (notched R) or the S wave (notched S) or the presence of more than one R' (fQRS) in two contiguous inferior (II, III, aVF), lateral (I, aVL, V6) or anterior (V1 to V5) leads. In ECG’s with a bundle branch block pattern or paced rhythms, fQRS was defined as more than two R waves (R'') or more than two notches in the R wave, or more than two notches in the downstroke or upstroke of the S wave[7,14]. Frontal plane QRS-T was calculated as the absolute difference between the QRS axis and the T axis, adjusted to an acute angle, for any calculation that was >180°. QRS-T angle was considered as abnormal if ≥90°. The following durations were considered prolonged if PR≥200ms, QRS≥120ms, and QTc≥470ms in females or ≥450ms in males[15]. The presence of a Q-wave was defined as >40ms in duration or >0.3 mV in depth (excluding the aVR) while criteria for a T-wave inversion included a negative amplitude of ≤1mm occurring in two neighbouring leads[16]. ST-segment elevation was classified if after the J point ≥0.1mV in females or ≥0.2mV in males the leads V2 and V3, or ≥0.1mV in other leads, and ST depression if was classified if a horizontal or downslope ≥0.1mV 0.08s after the J point along with a T-wave inversion ≥0.1mV in two contiguous leads[17]. An abnormal ECG was classified as having any one of the aforementioned ECG abnormalities.

### CMR imaging & analysis

Patient scanning was performed with a 3.0-T or a 1.5-T system (Tim Trio and Aera, Siemens, Erlangen, Germany). Detailed imaging information was reported previously[9,10]. All patients underwent standard cine imaging for acquisition of left and right ventricular mass and function, along with LGE imaging in matching slice locations. Patients were further categorized if the left ventricular end-diastolic volume index (LVEDV_i_) was enlarged in comparison to reference values[18], and LVEF was ≤50%. LGE presence was quantified visually and the extent of enhancement was quantified by using the full width half maximum signal intensity (FWHM) threshold cut-off and expressed as a percent of the LV myocardium[19]. From 2007, T2w inversion recovery images were included into the protocol and myocardial oedema was evaluated by assessing the ratio of the signal intensity in the LV myocardium compared to the skeletal muscle (musculus pectoralis major or minor). Since 2009, patients also underwent acquisition of T1 mapping and ECV calculations. Image analysis was performed with MASS v15 and QMASS MR (Medis Medical Imaging Systems, Leiden, the Netherlands).

### Clinical end-points

The primary clinical endpoint was time to the first major adverse cardiac events (MACE) from the CMR exam, which included a composite of all cause death, heart failure decompensation requiring hospital admission[20], heart transplantation, documented sustained ventricular tachycardia >30 seconds, or recurrent acute myocarditis. Recurrent myocarditis was based on the presentation of clinical symptoms, elevated biomarkers and CMR definitions of elevated T2w ratios and LGE enhancement in a non-ischemic pattern[3], occurring after recovery from the initial diagnosis. Follow-up data was obtained from electronic and medical records, or through contact with the patient.

### Statistical methods

Continuous variables were expressed as mean ± standard deviation while non-parametric values are reported as median values with interquartile range (IQR) and these variables were compared using a t-test or Wilcoxon rank-sum test, when appropriate. Categorical variables were presented as frequencies and percentages of the entire cohort or if relevant data were not available as a percentage of the corresponding group. These were compared using the Chi-square or Fisher exact test. Univariable and multivariable associations of risk covariates with clinical events were determined by Cox proportional hazards ratio (HR) regression and reported with 95% confidence intervals. To address ECG characteristics, two multi-variable models were formed to avoid statistical overfitting. The first model was imaging based and assessed a parsimonious set of variables composed of age, sex, LGE, LVEF and LVEDV_i_ with a single composite ECG variable. The second multivariable model was formed to investigate the individual ECG parameters, and this model included ECG characteristics that demonstrated a univariable association with MACE. Specific variables of age, sex, LGE and the presence of dilated left ventricle with reduced ejection fraction were added to this model. T2w images, along with T1 and ECV were not forwarded into the multi-variable models as they were added into the protocol part way through the study and were thus only available for a portion of the cohort. Kaplan Meier was used to plot cumulative incidence for MACE and compared using log-rank tests. Statistical significance was considered with a two-sided p-value of <0.05. IBM SPSS Statistics 26 (IBM, Armonk, NY, USA) and GraphPad Prism version 8.0 (GraphPad Software, La Jolla California USA) were used for all statistical analysis.

The study complies with the Declaration of Helsinki and the protocol was reviewed and approved by our Partners Human Research Committee (PHRC) Institutional Review Board in accordance with our institutional guidelines at the Brigham and Women's Hospital, Shapiro Cardiovascular Center, Boston, Massachusetts, United States, 02115. Informed consent was waived by the Review Board, and patients could refuse follow-up contact by returning a study letter.

## Results

### Population characteristics

**Fig 1:**
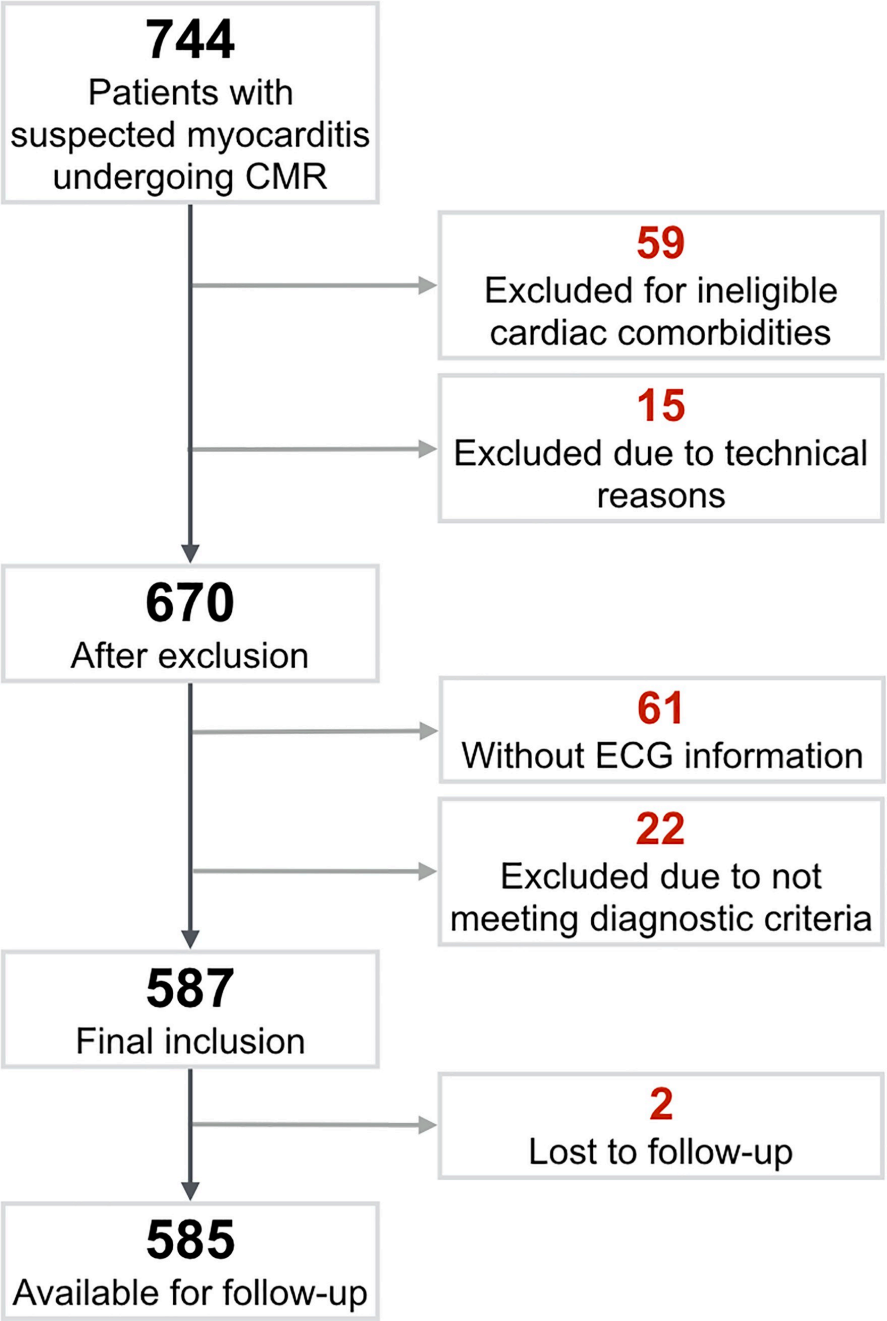
Patient enrolment. A total of 744 patients were referred to cardiovascular magnetic resonance for suspected myocarditis with 587 patients included in the final analysis.

From the initial population group[9], 587 patients were included, of which two were lost (0.3%) during follow-up leaving 585 for assessment with outcome (Fig 1, S1 Data.). The ECG was performed at a median of one day prior to the CMR exam [IQR: -6 to 0 days]. Baseline characteristics are displayed in Table 1. Of the entire population, 16% (n = 94) of the CMR exams were considered normal (i.e. no wall motion abnormalities, no LGE presence, no elevated T2w ratio in any segment and a LVEF>50%. Of these 94 patients, a further 26 patients had elevated troponins, while the remainder had abnormal ECG findings, fulfilling the diagnostic criteria[11]. Fifty-six patients with an absence of LGE also had normal troponin measurements.

**Table 1:** Patient characteristics.

Factor	All (n = 587)	LGE Absent (n = 312)	LGE Present (n = 275)	p-value
**Characteristics**				
Age (years)	48±16	47±15	49±15	0.149
Gender (females)	242 (41)	159 (51)	83 (30)	<0.001*
BMI (kg/m^2^)	27.7±6.2	27.4±6.1	27.9±6.0	0.348
**Medical History**				
Hypertension	153 (26)	81 (26)	72 (29)	0.907
Smoking	72 (12)	36 (12)	36 (13)	0.988
Diabetes Mellitus	53 (9)	32 (10)	21 (8)	0.441
Acute Presentation of Symptoms (<2 weeks)	323 (55)	161 (50)	162 (59)	0.081
Chest Pain	104 (17)	49 (16)	46 (17)	0.738
Dyspnoea	197 (32)	85 (27)	110 (40)	0.001*
Arrhythmia	117 (19)	75 (24)	42 (15)	0.009*
Palpitations	110 (18)	69 (22)	39 (14)	0.014*
Syncope	50 (8)	32 (10)	15 (5)	0.034*
Recent Infection	150 (35)	68 (22)	79 (29)	0.057
**Medication**				
ASA	165 (29)	77 (25)	88 (32)	0.061
ACE Inhibitors	208 (36)	100 (33)	108 (39)	0.057
Beta Blockers	236 (41)	102 (33)	134 (50)	<0.001*
Diuretics	122 (21)	47 (16)	75 (28)	<0.001*
Statins	123 (21)	53 (17)	70 (26)	0.011*
ARB	15 (3)	7 (2)	8 (3)	0.794

Mean±SD or n (%) are shown for the entire population and dichomotomized by the absence or presence of LGE. ACE: Angiotensin-converting enzyme inhibitors, ARB: Angiotensin II Receptor Blockers, ASA: Acetylsalicylic acid, BMI: Body Mass Index, LGE: Late Gadolinium Enhancement.

*p<0.05 denotes a significant difference between groups.

### ECG parameters

Seventy-eight percent of the population had an abnormal ECG at the time of the CMR exam (Table 2). One of the most frequent abnormalities observed was prolonged QTc duration (34%), along with an abnormal QRS-T angle (22%). fQRS complexes were present in 23% of the patients. When classifying the type of fQRS, the most common type was a notched R (15%) followed by notched S (11%), rSr (7%), RSR’ (4%) and fQRS (4%). Of the 132 patients with fQRS, a bundle branch block was present in 21 of these cases, and no significant association was observed between these two characteristics (p = 0.798). Characteristics such as ST-elevation, ST-depression and PR-depression were less prevalent, afflicting ≤5% of the patients.

**Table 2:** ECG and CMR findings and association with MACE.

		Univariable (MACE)	
Factor	Total	HR (95% CI)	p-value
**ECG**			
Abnormal ECG	457 (78)	2.20 (1.17–4.12)	0.014*
QRS-T Angle (°)	36 [17–83]	1.01 (1.01–1.01)	<0.001*
Wide QRS-T Angle (≥90°)	132 (22)	2.41 (1.59–3.66)	<0.001*
Low Voltage	51 (9)	1.87 (1.06–3.30)	0.031*
fQRS☨	132 (23☨)	0.76 (0.46–1.25)	0.279
*Notched R*	86 (15)	0.67 (0.36–1.27)	0.221
*Notched S*	60 (11)	0.89 (0.46–1.72)	0.731
*rSr*	38 (7)	0.86 (0.38–1.97)	0.722
*RSR’*	22 (4)	0.97 (0.31–3.05)	0.952
*Fragmented*	21 (4)	0.54 (0.13–2.20)	0.390
Q-wave	77 (13)	1.35 (0.79–2.32)	0.272
T-wave	175 (30)	1.29 (0.85–1.96)	0.240
ST Elevation	32 (5)	0.44 (0.14–1.40)	0.164
ST Depression	26 (4)	0.58 (0.18–1.84)	0.356
PR Depression	10 (2)	0.05 (0.00–21.5)	0.328
PR Duration (ms)	161±36	1.00 (0.99–1.00)	0.659
PR Duration (≥200ms)	43 (7)	1.33 (0.64–2.76)	0.443
QRS Duration (ms)	100±23	1.00 (0.99–1.01)	0.927
QRS Duration (≥120ms)	99 (17)	0.89 (0.51–1.58)	0.700
QTc Duration (ms)	445±49	1.01 (1.01–1.02)	<0.001*
QTc Duration (≥470 for females, ≥450 for males)	197 (34)	2.14 (1.42–3.21)	<0.001*
Left Bundle Branch Block	55 (9)	0.76 (0.35–1.63)	0.476
Right Bundle Branch Block	42 (7)	1.11 (0.54–2.29)	0.785
**Tissue Characterisation**			
LGE Presence	275 (47)	2.17 (1.43–3.31)	<0.001*
T2w	117 (28☨)	1.91(1.15–3.15)	0.012*
T1 (≥1072ms)	65 (40☨)	0.86 (0.35–2.12)	0.753
ECV (≥35%)	45 (20☨)	3.43 (1.42–8.29)	0.006*
**Characteristics**			
LVEF (%)	47.9±15.6	0.96 (0.95–0.97)	<0.001*
LVEDV_i_ (ml/m^2^)	99±34	1.01 (1.00–1.01)	0.001*
Dilated LVEDV_i_ & LVEF≤50%^**‡**^ • >95 ml/m^2^ (Females <60years) • >86 ml/m^2^ (Females ≥60years) • >100 ml/m^2^ (Males <60years) • >94 ml/m^2^ (Males ≥60years)	165 (28)	2.74 (1.77–4.24)	<0.001*
LVESV_i_ (ml/m^2^)	55±36	1.01 (1.01–1.02)	<0.001*
CI (ml/min/m^2^)	3135±816	1.00 (0.99–1.00)	0.004*
LV Mass_i_ (g/m^2^)	61±17	1.01 (1.00–1.03)	0.073
RVEF (%)	48.5±11.4	0.95 (0.93–0.96)	<0.001*
Pericardial Effusion	157 (27)	2.15 (1.43–3.24)	<0.001**
Pleural Effusion	78 (13)	3.86 (2.48–6.01)	<0.001*
Age (years)	48±16	1.03 (1.01–1.04)	<0.001*
Sex (female)	242 (41%)	1.51 (1.01–2.27)	0.044*
BMI (kg/m^2^)	27.7±6.1	1.05 (1.02–1.09)	0.001*
**Peak Blood Markers**^**§**^			
Creatinine Kinase (U/l)	140 [69–506]	1.00 (1.00–1.00)	0.844
C-reactive protein (mg/dl)	13 [4–74]	1.00 (0.99–1.01)	0.884
NT-proBNP (ng/ml)	1.51 [0.31–5.20]	1.13 (1.08–1.17)	<0.001*
Troponin (ng/ml)	0.08 [0.00–0.45]	1.10 (0.92–1.31)	0.311
White blood cell count (10^9^/l)	8.4 [6.7–11.6]	1.01 (0.97–1.06)	0.581

Mean±SD, median [interquartile range] or n (%) are shown for the ECG and CMR findings, along with the univariable hazard ratio and 95% confidence intervals for the association with MACE (total of 94 events). BMI: Body Mass Index, CI: Cardiac Index, ECV: Extracellular Volume, HR: Hazard Ratio, LGE: Late Gadolinium Enhancement, LVEF: Left Ventricular Ejection Fraction, LVEDV_i_: Left Ventricular End Diastolic Volume Index, LVESV_i_: Left Ventricular End Systolic Volume Index, RVEF: Right Ventricular Ejection Fraction, T2w: T2 Weighted.

*p<0.05 denotes a significant univariate relationship.

☨fQRS analysis was only available in 571 participants, T2 in 425 cases, T1 in 164 cases and ECV in 153.

‡Cut-off measurements for LVEDV_i_ based on +two standard deviations of age-stratified reference values[18].

§Laboratory markers were not systemically measured in all patients and were available in less than half the population.

### Association of ECG and CMR tissue characterisation

**Fig 2:**
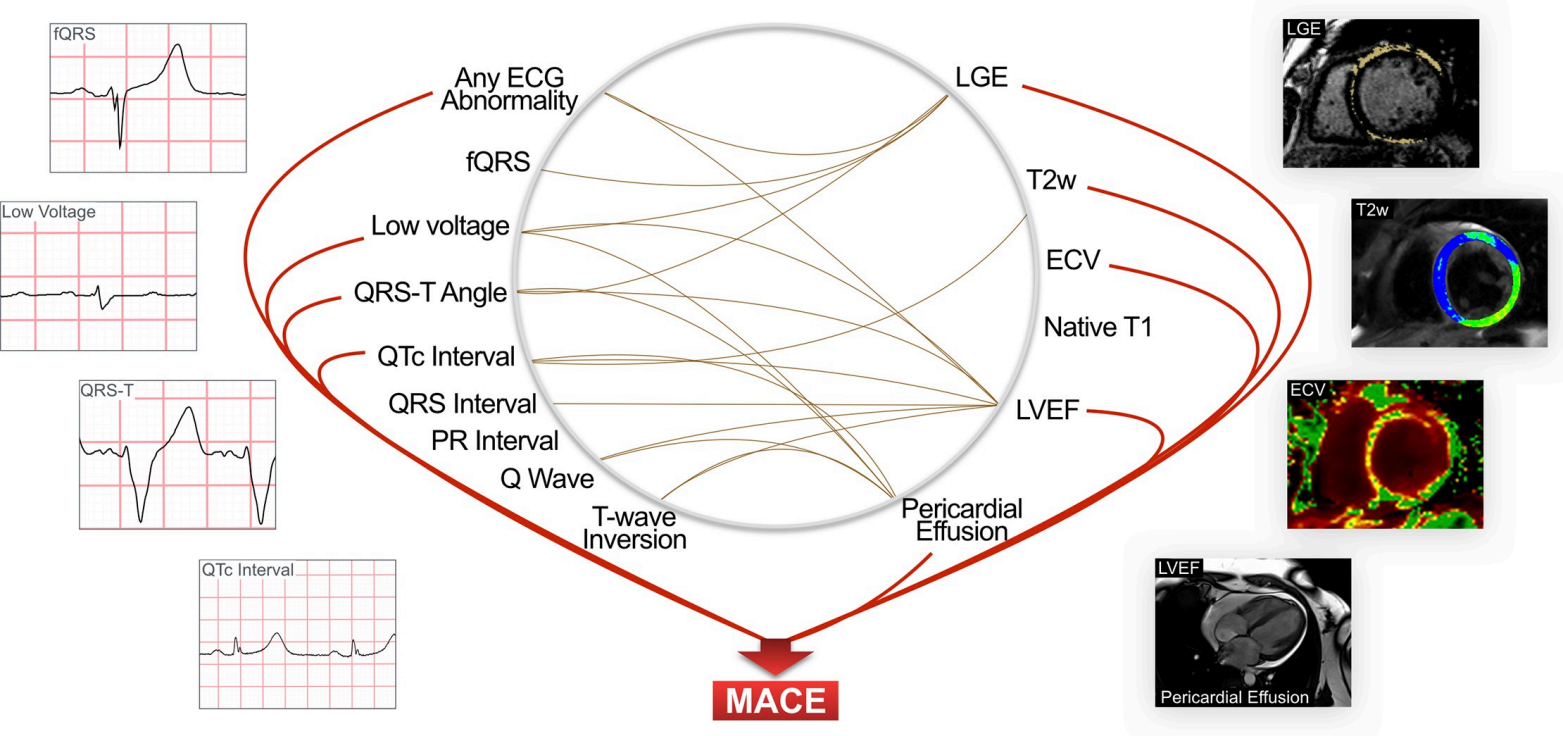
Relationship between ECG and CMR parameters and their association with MACE. Brown connecting lines indicate significant associations (p<0.05) between CMR parameters and ECG parameters. Red lines represent significant associations (p<0.05) with MACE. The presence of an ECG abnormality was associated with a greater prevalence of patients with LGE, oedema or pericardial effusion, or with a lower ejection fraction. ECV: Extracellular Volume, LGE: Late Gadolinium Enhancement, LVEF: Left Ventricular Ejection Fraction, MACE: Major Adverse Cardiac Event, T2w: T2 Weighted.

Forty-seven percent of this cohort had LGE with a mean extent of 2.9±5.6%. When comparing the presence of LGE to ECG findings, a greater prevalence of patients had LGE in the presence of an abnormal ECG (50%), a wide QRS-T angle (58%), with low voltage QRS (61%), and fQRS (62%) being associated with the highest proportion of LGE (Table 3). T1 and ECV measurements were available in 164 and 153 patients, of whom 40% (n = 65) and 20% (n = 40) had a globally elevated native T1 and ECV respectively. There was no association of native T1 with any ECG parameter (Fig 2). Categorically, a non-significant trend p = 0.067 was observed between ECV and fQRS (S1 Table). Twenty-eight percent of the population had a globally elevated T2w-ratio at the time of imaging, while 75% of patients with T2w images showed T2w enhancement in at least one segment. Elevated T2w was more prevalent when there was an elongated QTc duration.

**Table 3:** Association of ECG parameters to LGE presence and abnormal T2w.

Factor	LGE presence					T2w				
	ECG Factor Absent	ECG Factor Present	Sensitivity	Specificity	p	ECG Factor Absent	ECG Factor Present	Sensitivity	Specificity	p
Abnormal ECG	47 (36)	228 (50)	83 (78–87)	27 (22–32)	0.006*	23 (23)	94 (30)	80 (72–87)	25 (21–31)	0.215
Wide QRS-T Angle (≥90°)	197 (44)	77 (58)	28 (23–34)	82 (78–86)	0.003*	84 (26)	31 (33)	30 (20–36)	79 (74–83)	0.180
Low Voltage	244 (46)	31 (61)	11 (8–16)	94 (90–96)	0.039*	101 (27)	16 (41)	14 (9–21)	93 (89–95)	0.061
fQRS	187 (43)	82 (62)	30 (25–36)	83 (79–87)	<0.001*	90 (28)	25 (28)	22 (15–30)	78 (73–83)	0.989
Q-wave	234 (46)	41 (53)	15 (11–20)	88 (84–92)	0.233	95 (27)	21 (36)	18 (12–26)	88 (83–91)	0.131
T-wave	187 (46)	86 (49)	32 (26–37)	71 (66–76)	0.448	78 (27)	37 (31)	32 (24–41)	72 (67–77)	0.349
PR Duration (≥200ms)	230 (46)	24 (56)	9 (6–14)	94 (90–96)	0.225	102 (29)	7 (24)	6 (3–13)	92 (88–95)	0.604
QRS Duration (≥120ms)	236 (48)	39 (40)	14 (11–19)	81 (76–85)	0.103	96 (28)	21 (28)	18 (12–26)	82 (77–86)	0.916
QTc Duration (≥470ms for females, ≥450ms for males)	174 (45)	101 (51)	37 (31–43)	69 (64–74)	0.127	67 (24)	50 (35)	43 (34–52)	69 (64–74)	0.021*

Frequency and (%) is shown for the number of patients who were categorically classified as having Late Gadolinium Enhancement (LGE) or an elevated T2-weighted (T2w) in the absence and presence of ECG characteristics. Sensitivity and specificity with 95% confidence intervals are shown for the ECG parameters to correctly identify patients with LGE or elevated T2w. fQRS: Fragmented QRS

*p<0.05 denotes a significant relationship.

Pericardial effusion was present in 27% of the patients, the proportion of ECG features T-wave inversion (25 vs 42%, p<0.001), low voltage (7 vs 14%, p = 0.005), abnormal Q-wave (11 vs 19%, p = 0.019), QTc duration (30 vs 43%, p = 0.005) and a wide QRS-T angle (18 vs 36%, p<0.001) were greater when pericardial effusion was present. The associations of ECG with pericardial effusion and ejection fraction are shown in Table 4, with additional volumetric data presented in S2 Table in patients with and without LGE enhancement.

**Table 4:** Association of ECG parameters to pericardial effusion and LVEF.

Factor	Pericardial Effusion					LVEF<40%				
	ECG Factor Absent	ECG Factor Present	Sensitivity	Specificity	p	ECG Factor Absent	ECG Factor Present	Sensitivity	Specificity	p
Abnormal ECG	27 (21)	130 (28)	83 (76–88)	24 (20–28)	0.081	15 (12)	174 (38)	92 (87–95)	29 (25–33)	<0.001*
Wide QRS-T Angle (≥90°)	100 (22)	57 (43)	36 (29–44)	82 (78–86)	<0.001*	106 (24)	81 (61)	43 (36–50)	88 (83–90)	<0.001*
Low Voltage	134 (25)	22 (43	14 (9–20)	93 (90–95)	0.005*	164 (31)	24 (47)	13 (7–18)	94 (91–96)	0.017*
fQRS	122 (28)	31 (23)	20 (14–27)	76 (72–80)	0.327	147 (33)	37 (28)	20 (15–26)	75 (71–80)	0.234
Q-wave	127 (25)	29 (38)	18 (13–25)	89 (86–91)	0.019*	149 (29)	40 (52)	21 (16–28)	91 (87–93)	<0.001*
T-wave	90 (22)	66 (38)	42 (35–50)	75 (70–78)	<0.001*	119 (29)	69 (39)	37 (30–44)	73 (69–77)	0.014*
PR Duration (≥200ms)	136 (27)	7 (16)	5 (2–10)	91 (88–93)	0.116	149 (30)	14 (33)	9 (5–14)	92 (89–95)	0.718
QRS Duration (≥120ms)	126 (26)	31 (31)	20 (14–27)	84 (80–87)	0.260	132 (27)	57 (58)	30 (24–37)	89 (86–92)	<0.001*
QTc Duration (≥470ms for females, ≥450ms for males)	90 (23)	67 (34)	42 (35–51)	69 (65–74)	0.001*	74 (19)	115 (58)	61 (54–68)	79 (75–83)	<0.001*

Frequency and (%), along with sensitivity and specificity and 95% confidence are shown for the number of patients who were categorically classified with pericardial effusion and with left ventricular ejection fraction (LVEF) below 40% based on the absence or presence of ECG parameters. ECG: Electrocardiogram, fQRS: Fragmented QRS.

*p<0.05 denotes a significant relationship.

### Association of ECG and CMR parameters with MACE

After a median follow-up time of 3.9 years (IQR: 1.8–6.9), 94 patients were documented to have MACE, including 27 deaths, 38 patients hospitalised for heart failure, 1 cardiac transplant, 22 cases of sustained arrhythmia and 6 cases of recurrent myocarditis as the first MACE. In a cox-proportion HR regression analysis, univariable tests demonstrated that for the ECG parameters only an abnormal QRS-T angle, low voltage QRS and QTc duration were associated with adverse outcomes, along with an overall abnormal ECG (Table 2, Fig 2). Additionally we investigated a composite variable that was considered positive if a patient presented with at least one of the following specified ECG variables; fQRS, a wide QRS-T angle, low QRS voltage or a prolonged QTc interval, and this variable yielded a HR of 2.67 (1.61–4.42), p<0.001. For CMR features, the presence of LGE was strongly associated with MACE. Furthermore, both an abnormal ECV≥35% representing focal and diffuse fibrosis and the presence of an elevated T2w ratio ≥2, a marker of oedema demonstrated prognostic value in addition to multiple demographic and ventricular function markers. Laboratory blood markers of troponins and creatinine-kinase were not associated with a higher risk of MACE. NT-proBNP was linked with MACE, although it was only assessed in 132 patients.

**Fig 3:**
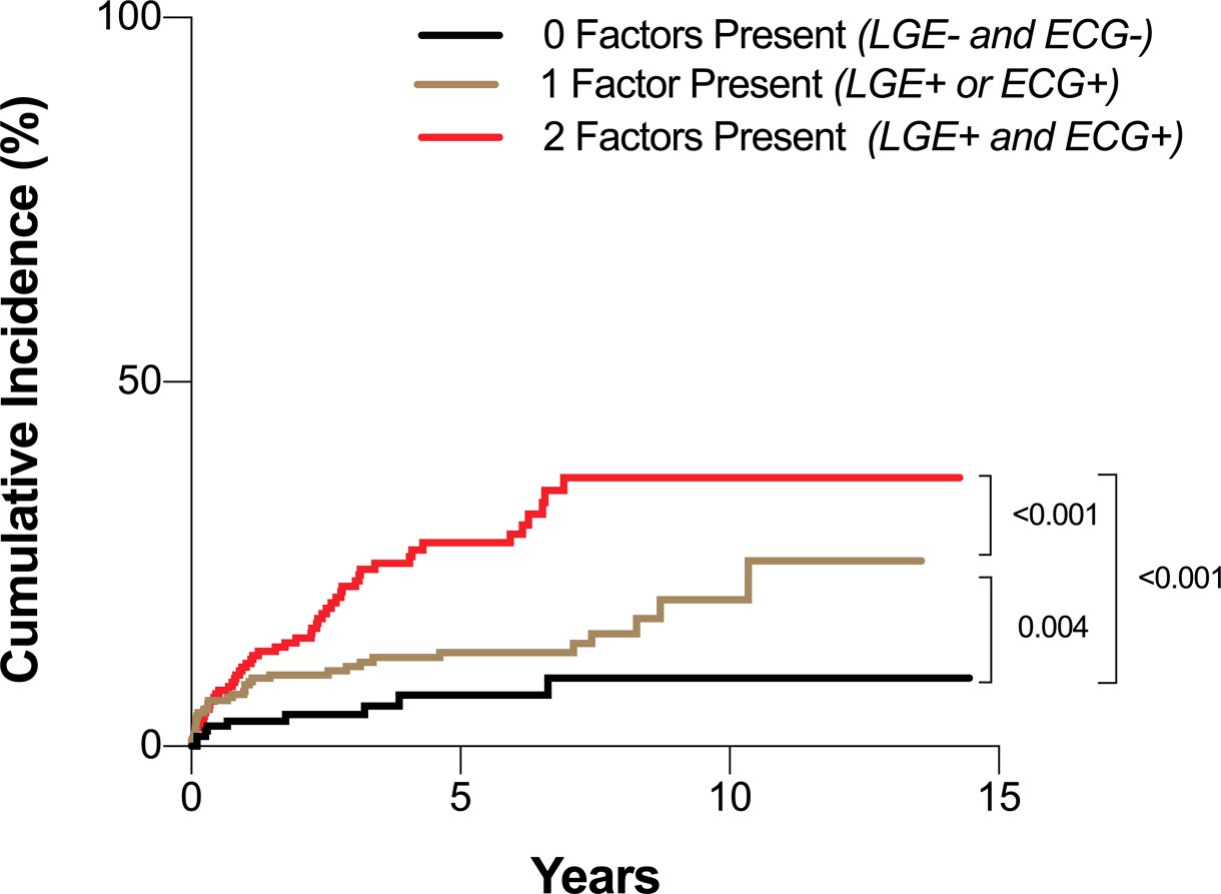
Cumulative incidence of MACE. Kaplan Meier curve of cumulative incidence, based on the presence of late gadolinium enhancement (LGE) and an abnormal electrocardiogram (ECG) marker of either fragmented QRS, wide QRS-T angle, prolonged QTc duration or low QRS voltage.

**Fig 4:**
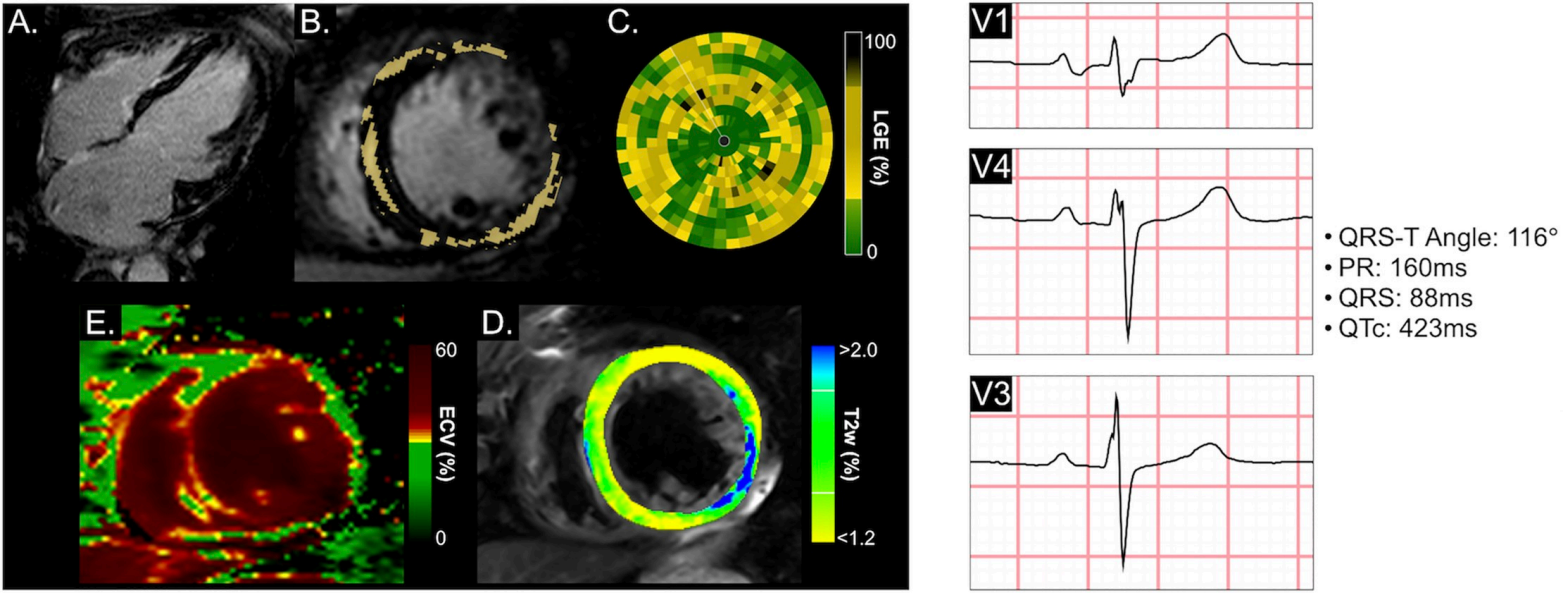
Patient with sustained ventricular tachycardia. This is a case of a 62 year old male who experienced sustained ventricular tachycardia 4 months following the CMR exam, followed by death after 3.0 years. LGE Images (A-C) show a midwall distribution and linear pattern in the long-axis view (A), of which the extent of LGE was 11.0% highlighted yellow in the short-axis view (B). A bullseye plot representing the full left ventricle, shows the relative enhancement (yellow and black) afflicted all ventricular walls. The ECV map (D) demonstrated a similar pattern with a global ECV of 39.8% (red), while some oedema in the lateral wall (blue) was detected in the T2w images (E). At the CMR exam, left ventricular ejection fraction was 40%. A wide QRS-T angle was detected in the ECG. ECV: Extracellular Volume, fQRS: Fragmented QRS, LGE: Late Gadolinium Enhancement, T2w: T2 Weighted.

In the first multivariable model (Table 5), all CMR measurements (LGE, LVEF, and LVEDV_i_) remained significantly independently associated with outcome, along with the composite ECG variable (fQRS, QRS-T angle, low QRS voltage, or QTc). In the second multivariable model investigating individual ECG parameters, LGE, a wide QRS-T-angle and a low QRS voltage remained significantly independently associated with outcome (Table 5). Furthermore, as shown in Fig 3, the cumulative incidence of MACE was higher in patients where LGE was present or when there was an abnormality in one of the ECG markers of fQRS, wide QRS-T angle, prolonged QTc duration or low voltage (p = 0.019). This was even greater when both LGE and these specific ECG abnormalities were present (p<0.001). This can be observed in two patient cases (Fig 4 and S1 Fig).

**Table 5:** Multivariable analysis for outcome prediction.

	HR (95% CI)	p-value
**Model 1**		
Age (years)	1.02 (1.00–1.03)	0.038*
Sex	1.52 (0.97–2.36)	0.066
LGE	1.69 (1.05–2.72)	0.033*
LVEDV_i_ (ml/m^2^)	0.99 (0.98–1.00)	0.039*
LVEF (%)	0.95 (0.93–0.97)	<0.001*
Composite ECG of either • Wide QRS-T Angle • Low Voltage • Prolonged QTc Duration • fQRS	1.82 (1.02–3.24)	0.042*
**Model 2**		
Age (years)	1.02 (1.00–1.03)	0.037*
Sex	1.65 (1.05–2.59)	0.030*
LGE	1.90 (1.17–3.10)	0.010*
Dilated LVEDV_i_ & LVEF≤50%	1.62 (0.98–2.63)	0.052
QRS-T Angle (°)	1.01 (1.00–1.01)	0.029*
Low Voltage	1.86 (1.01–3.42)	0.046*
QTc Duration (≥470ms for females, ≥450ms for males)	1.51 (0.93–2.47)	0.099

Two different multivariable models incorporating demographics, CMR and ECG parameters. HR: Hazard Ratio, LGE: Late Gadolinium Enhancement, LVEDV_i_: Left Ventricular End-Diastolic Volume Index, LVEF: Left Ventricular Ejection Fraction.

*p<0.05 denotes significance.

As shown in the S3 Table, patients with acute presentation of symptoms were more likely to present with an abnormal ECG (85% vs 69%, p<0.001), and a longer QTc duration, but no differences were observed with QRS-T angle, low QRS voltage, LVEF or the CMR tissue characterisation sequences of LGE, T2w. In this subset with acute symptoms, all these parameters maintained univariable significance for the association of MACE, while in the group with subacute presentation, QRS-T angle, LGE, ECV and LVEF were significant.

## Discussion

In the present study, ECG parameters were associated with abnormal CMR tissue characterisation in patients with suspected myocarditis. Besides LGE presence in CMR images, a wide QRS-T angle and low QRS voltage showed independent prognostic information in this cohort. Further, a combination of both ECG parameters with LGE findings showed incremental value for prognostication in patients with suspected myocarditis.

### ECG characteristics as markers of myocardial injury

As myocarditis may present with various signs and symptoms, integrating multiple tests into clinical decision-making can amplify the likelihood of detecting prognostic factors and may therefore help in risk stratifying these patients. While the Lake Louise Criteria propose CMR as the primary modality to detect signs of acute myocardial inflammation and other markers of myocardial injury associated with myocarditis[3,21], ECG is not implemented in these recommendations as a stand-alone exam. We could demonstrate that ECG abnormalities are linked to myocardial injury as shown by LGE and oedema in CMR and also independently to outcome. This may be beneficial as upon suspicion of myocarditis, the majority of patients will undergo a routine ECG recording in their clinical follow-up. Moreover, ECG is a fast and cheap non-invasive test, which is also available outside of tertiary centres compared to CMR.

ECG abnormalities have been previously associated with LGE in many cardiovascular disorders[7,22]. Underlying structural abnormalities such as fibrosis and oedema impact electrical homogeneity across the heart leading to conduction delays, resulting in abnormal ECG findings. These are not isolated to significant transmural enhancement, but other patterns such as diffuse fibrosis in left ventricular hypertrophy[23]. Specifically for myocarditis, it was demonstrated in 65 patients with biopsy-proven myocarditis that 77% had an abnormal ECG, similar to our findings that 78% of the population had an abnormal ECG, and this was associated with LGE findings where bundle branch block was observed with septal enhancement, ST-abnormalities with lateral enhancement, while transmural LGE in the lateral wall was associated with Q-waves[24]. On the other hand, a separate study linked T-wave inversion with myocardial oedema in 76 acute myocarditis patients[16]. Similarly, we could show an association of T2w imaging and pericardial effusion, which could be an indirect sign of inflammation of the heart muscle and pericardium. In the case of suspected myocarditis, the presence of certain characteristics found in the ECG indicate a greater likelihood of oedema and fibrosis or necrosis, known markers of myocarditis[3,21]. Here we also observed that LGE was significantly more prevalent in the case of an abnormal ECG in comparison to a normal reading. Specifically, QRS-T angle, low QRS voltage and fQRS were all significantly associated with LGE. The highest proportions of patients with LGE were seen with abnormalities in the QRS complex, when fQRS or low voltage QRS were present. However, in this population all with suspected myocarditis 78% had an abnormal ECG, still leaving 22% with normal readings. Similarly, Di Bella *et al*, found in 81 acute myocarditis patients positive for LGE and 68% had abnormal ECG findings[25]. Consequently, a normal ECG is not promising as a technique for ruling out myocardial injury in comparison to CMR of patients with suspected myocarditis. Thus, CMR plays a superior role for diagnosing and characterising myocarditis[3,21]. Furthermore, one of CMR’s principal advantages is that not only can it indicate the presence of fibrosis, necrosis or oedema as a binary variable, but observation of the pattern, location and size can aid in distinguishing myocarditis from other cardiovascular disease and provide further information on the severity of myocardial damage

### Predictors of outcome

Our findings of the association of ECG abnormalities with the development of MACE supports previous publications in smaller samples of myocarditis patients. In a study without using CMR, QRS-T angle was found to be a predictor of heart failure and combined heart failure and death in 193 myocarditis patients[26]. In our analysis, an abnormal ECG, a wide QRS-T angle, low voltage, and QTc duration were all predictors of outcome from the univariate analysis. QRS-T angle and low QRS voltage were the only ECG variables remaining as independent outcome predictors in the multivariable model. This can likely be explained by the fact that a wider QRS-T angle can reflect a mismatch between ventricular de- and repolarisation, which can be the source for potentially ventricular arrhythmias and consecutive adverse cardiac events. Reflecting the known variability of myocarditis, other studies have shown in line with our study that different ECG parameters have prognostic value including QRS and QTc prolongation, ST elevation and presence of Q-wave[27,28]. These different findings between studies can be impacted by patient population, size, and importantly variables included into the statistical models especially considering few incorporate both CMR and ECG findings together. Considering, multiple ECG parameters have been implicated with morbidity and mortality, we also assessed the impact of a generally abnormal ECG, not specific to the exact ECG parameter. However, many of these aforementioned ECG abnormalities that are linked to myocarditis are also associated with other poor outcome in other cardiovascular diseases, and thus is not discriminatory solely to myocarditis[7,17,29].

The prognostic potential of LGE and ECV in this population of suspected myocarditis has been previously demonstrated[9,10] and in multiple publications[30,31] including in an independent study with 222 patients where LGE was shown to be the superior prognostic factor for cardiac death and all-cause mortality in comparison to other CMR functional measurements including LVEF[32]. Similarly, in the current analysis multiple CMR markers were associated with MACE in the univariate model, including pericardial effusion, multiple left ventricular volumes, LVEF and cardiac index, along with the tissue characterisation measures of LGE, T2w imaging and ECV. As the multi-variate analysis showed both CMR and ECG markers have strong independent prognostic ability, and consequently a combination of both markers yielded a significantly higher incidence rate. Thus, the incorporation of ECG into the CMR assessment may yield additional value when assessing the future outcome in the patient when myocarditis is determined.

### Presentation at time of CMR and ECG exams

Findings from diagnostic exams may be confounded by the timing of the exam in relation to the onset of myocarditis and symptoms. ECG characteristics may be more transient appearing and then normalizing with different stages of myocarditis[33], and in our analysis some features are more present in patients with acute presentation. To a similar extent, myocardial oedema may also not be as persistent, and a positive finding can be time dependent based on if imaging is performed during a stage of active inflammation. It has been independently reported that T2 mapping could discriminate between acute and recovered stages of myocarditis, because T2 regressed by 5 weeks and nearly normalized within 6 months, while measurements of fibrosis did not change over time[34]. In our population, there was no difference in the T2w ratio between patients with acute (<2 weeks) and sub-acute presentation (2–4 weeks), but T2w imaging had a univariable prognostic role in patients with acute presentation of symptoms and not in sub-acute. On the other hand, LGE and ECV indicate more permanent damage and can be observed at various stages of disease progression. Consequently, both of these parameters demonstrated prognostic value in our sub-acute patients as well. In this analysis, we present measurements from a single ECG and CMR, repetitive examinations would better demonstrate if these findings are stable or temporary.

### Limitations

First, our study has the limitations from a retrospective design without a strategic randomisation to any specific therapy. Consequently, potential biases introduced by CMR and ECG findings to patient outcomes due to medical or procedural therapies exist. Second, this was a study where imaging was performed over a duration of nearly fourteen years with a modality that is rapidly developing. While all patients underwent a consistent protocol of LGE imaging and cine imaging for the assessment of ventricular function and morphology, additional sequences for tissue characterisation such as T2w imaging were only added when they became available to imaging centres, consequently statistical findings with these sequences are limited by smaller sample size. Additionally, because of this extended time-frame, there was no consistent validated imaging gold-standard for the diagnosis of myocarditis at the time of presentation. For example, the initial version of the Lake Louise Criteria was not released until 2009[3], already seven years after the first patient. Furthermore, of the three criteria listed in the first iteration, only LGE was always available, with T2 imaging not being developed until later in the study, and early gadolinium enhancement not being routinely performed clinically on site, consequently retrospective assessment of Lake Louise Criteria could not be applied. Similarly, endomyocardial biopsy was only performed for 56 (9%) of the patients based on the treating clinician’s discretion, and consequently was not included into the data analysis for this study. While considered a gold-standard marker, biopsy use is infrequent and can be prone to sampling error. Consequently, we applied guidelines from working groups of the European Society of Cardiology supported by the American Heart Institute, which includes multiple non-invasive biomarkers and imaging features[11,12]. These diagnostic criteria are more compatible with the data available for this cohort, as they are not solely dependent on tissue characterization, but also include clinical diagnosis when tissue characterization may not be available. Thus, this population was selected by a suspicion of myocarditis through clinical presentation and compatible diagnostic features, along with a ruling out process, where patients were excluded if other diagnostic testing indicated a high likelihood for a different comorbidity. This method also reflects the clinical methods of myocarditis diagnosis at the time of recruitment. However, it is likely our population includes patients who may not have myocarditis. Additionally, we do not distinguish patients with dilated cardiomyopathy, although myocarditis is a known underlying cause of dilated cardiomyopathy. Even using modern diagnostic techniques, accurate diagnosis of myocarditis is limited[35]. It is important to note that our study did not investigate the diagnostic accuracy of these techniques, rather compared the association of ECG and CMR findings in a real-world setting of patients with a clinical referral of suspected myocarditis. Nevertheless, our results show that in patients where myocarditis is suspected even in the absence of a definitive diagnosis, specific ECG parameters are related to CMR findings, and these markers have a prognostic role.

On a technical component, T2w images are often affected by artifacts which may introduce inaccuracies to T2w ratio, and these ratios used for quantitative assessment are based on the assumption of a healthy skeletal muscle. Technical development of T2 mapping may be promising in scaling the severity of myocardial oedema and for detecting low levels of inflammation, but these were not available in this patient cohort. LGE is a sequence that detects only regional enhancement within the myocardium. Therefore, newer techniques, such as T1 mapping and ECV may overcome this limitation by providing quantitative information on both focal and diffuse fibrosis. In this cohort, ECV were also associated with outcome with univariable analysis, and ECG parameters despite lower sample sizes and events. Similarly, newer high-sensitive assays for blood markers that were not available for this population may have better prognostic potential as well and in our analysis laboratory blood tests were performed in less than half the participants, on the discretion of the treating clinician.

### Clinical implications

Our findings show that abnormal ECG parameters, specifically fQRS, a wide QRS-T angle and low voltage are associated with LGE, and their presence on an ECG can support initiation of a CMR exam in patients with the clinical suspicion of myocarditis. Further, the presence of a widened QRS-T angle or low QRS voltage depicted from ECG or LGE from CMR can be utilised to identify the potential risk of future MACE. This is one of the largest follow-up studies of this population where follow-up patient data was available up to 14 years after the initial CMR. As ECG is one of the routine clinical work-ups for suspected myocarditis, information of ECG, will likely be available more often than CMR findings. CMR is the recommended procedure for the confirmation of myocarditis as a more discriminatory exam, after which findings of myocardial injury can be included into the decision pathway of patient treatment.

## Conclusion

In patients with clinically suspected myocarditis, ECG abnormalities are associated with abnormal tissue characteristics from CMR. Further, both CMR and ECG findings have independent prognostic implications for morbidity and mortality. Integrating both exams into clinical decision-making may play a role in risk stratification in this heterogeneous patient population.

## Supporting information

S1 Fig Patient with recurrent myocarditis. A 35 year old female patient who experienced recurrent myocarditis 4.1 years following the CMR exam showed a predominantly patchy pattern and epicardial distribution of late gadolinium enhancement (LGE, A, B). The extent of LGE was 7.6% highlighted yellow in the short-axis view (B), primarily in the anteroseptal and anterolateral walls as shown by yellow and black in the bullseye plot. The extracellular volume (ECV) map (D) demonstrated a similar pattern of focal fibrosis, and additional diffuse fibrosis in the septum (44.3% globally), with a high T2-ratio in the anterior and septal walls (blue, E). At the CMR exam, left ventricular ejection fraction was 50%. This patient had fragmented QRS complex’s both at the initial CMR exam, and while not part of the analysis, fragmented (f)QRS was also present when the patient experienced recurrent myocarditis.
(PDF)

S1 Table Association of ECG parameters to T1 and ECV. Frequency and (%), along with sensitivity and specificity and 95% confidence are shown for the number of patients who were categorically classified with a native T1≥1072ms and an ECV≥35% based on the absence or presence of ECG parameters. ECG: Electrocardiogram, ECV: Extracellular Volume, fQRS: Fragmented QRS.
(PDF)

S2 Table Supplemental volumetric findings by LGE presentation. Mean±SD or n (%) are shown for ventricular volumes based on the absence or presence of Late Gadolinium Enhancement (LGE).
(PDF)

S3 Table Findings in acute and sub-acute symptom presentation. Mean±SD, median [interquartile range] or n (%) are shown for the ECG and CMR findings, along with the univariable hazard ratio and 95% confidence intervals for the association with MACE for patients with acute presentation of symptoms (65 events) vs patients with sub-acute presentation (29 events).
(PDF)

S1 Data Supporting data file. (XLSX)
