# Supplementary material for: Association of ECG parameters with late gadolinium enhancement and outcome in patients with clinical suspicion of acute or subacute myocarditis referred for CMR imaging
Source: PLoS One. 2020 Jan 10;15(1):e0227134. doi: 10.1371/journal.pone.0227134 (PMC6953836; doi:10.1371/journal.pone.0227134)
Supplement: S1 Fig — A 35 year old female patient who experienced recurrent myocarditis 4.1 years following the CMR exam showed a predominantly patchy pattern and epicardial distribution of late gadolinium enhancement (LGE, A, B). The extent of LGE was 7.6% highlighted yellow in the short-axis view (B), primarily in the anteroseptal and anterolateral walls as shown by yellow and black in the bullseye plot. The extracellular volume (ECV) map (D) demonstrated a similar pattern of focal fibrosis, and additional diffuse fibrosis in the septum (44.3% globally), with a high T2-ratio in the anterior and septal walls (blue, E). At the CMR exam, left ventricular ejection fraction was 50%. This patient had fragmented QRS complex’s both at the initial CMR exam, and while not part of the analysis, fragmented (f)QRS was also present when the patient experienced recurrent myocarditis. (PDF) [file pone.0227134.s001.pdf]

**S1 Fig. Patient with recurrent myocarditis.**

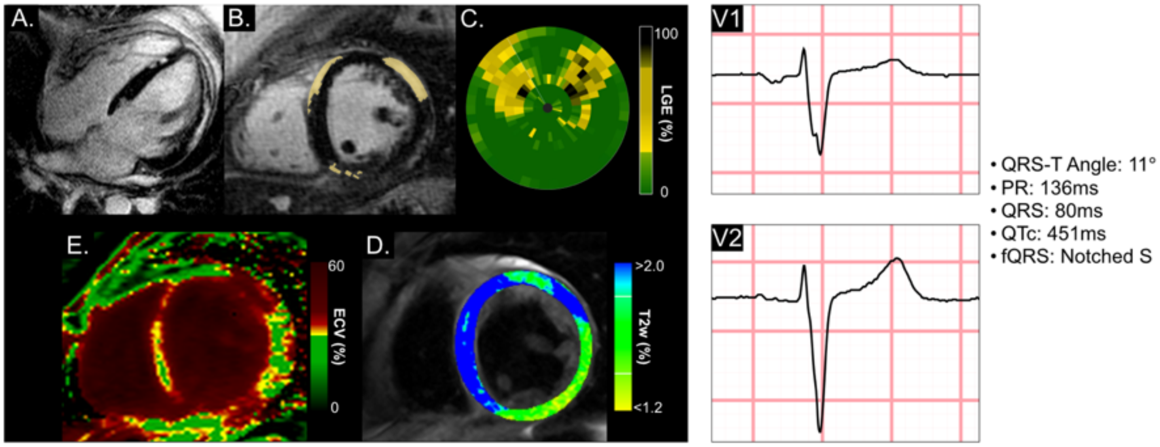


A 35 year old female patient who experienced recurrent myocarditis 4.1 years following the CMR exam showed a predominantly patchy pattern and epicardial distribution of late gadolinium enhancement (LGE, A, B). The extent of LGE was 7.6% highlighted yellow in the short-axis view (B), primarily in the anteroseptal and anterolateral walls as shown by yellow and black in the bullseye plot. The extracellular volume (ECV) map (D) demonstrated a similar pattern of focal fibrosis, and additional diffuse fibrosis in the septum (44.3% globally), with a high T2-ratio in the anterior and septal walls (blue, E). At the CMR exam, left ventricular ejection fraction was 50%. This patient had fragmented QRS complex's both at the initial CMR exam, and while not part of the analysis, fragmented (f)QRS was also present when the patient experienced recurrent myocarditis.
