# Supplementary material for: Association of ECG parameters with late gadolinium enhancement and outcome in patients with clinical suspicion of acute or subacute myocarditis referred for CMR imaging
Source: PLoS One. 2020 Jan 10;15(1):e0227134. doi: 10.1371/journal.pone.0227134 (PMC6953836; doi:10.1371/journal.pone.0227134)
Supplement: S1 Table — Frequency and (%), along with sensitivity and specificity and 95% confidence are shown for the number of patients who were categorically classified with a native T1≥1072ms and an ECV≥35% based on the absence or presence of ECG parameters. ECG: Electrocardiogram, ECV: Extracellular Volume, fQRS: Fragmented QRS. (PDF) [file pone.0227134.s002.pdf]

S1 Table. Association of ECG parameters to T1 and ECV.

Factor	Native T1 ≥1072 (ms)					ECV ≥35 (%)				
	**ECG Factor Absent**	**ECG Factor Present**	**Sensitivity**	**Specificity**	**p**	**ECG Factor Absent**	**ECG Factor Present**	**Sensitivity**	**Specificity**	**p**
Abnormal ECG	12 (30)	56 (42)	82 (72-90)	27 (19-36)	0.159	11 (26)	37 (31)	77 (63-87)	27 (20-36)	0.551
Wide QRS-T Angle (≥90ms)	52 (39)	15 (42)	22 (14-34)	80 (71-86)	0.755	37 (29)	9 (27)	20 (11-33)	79 (70-85)	0.814
Low Voltage	63 (40)	5 (36)	7 (3-16)	91 (84-95)	0.760	41 (28)	7 (50)	15 (7-27)	94 (88-97)	0.084
fQRS	50 (40)	16 (37)	24 (16-36)	73 (64-81)	0.719	31 (26)	14 (38)	31 (20-46)	79 (71-86)	0.067
Q-wave	59 (39)	9 (45)	13 (7-23)	89 (82-94)	0.611	40 (29)	8 (38)	17 (9-30)	88 (82-93)	0.385
T-wave	51 (39)	17 (41)	25 (16-37)	77 (68-84)	0.799	37 (30)	11 (30)	23 (13-37)	77 (68-84)	0.967
PR Duration (≥200ms)	62 (41)	3 (27)	5 (1-13)	92 (84-96)	0.359	44 (31)	2 (20)	4 (1-15)	92 (86-96)	0.449
QRS Duration (≥120ms)	52 (38)	16 (43)	24 (15-35)	80 (71-86)	0.603	39 (30)	9 (28)	19 (10-32)	80 (71-86)	0.816
QTc Duration (≥470 for females, ≥450 for males)	40 (37)	28 (44)	41 (30-53)	65 (56-74)	0.384	34 (33)	14 (24)	29 (18-43)	60 (51-69)	0.199

Frequency and (%), along with sensitivity and specificity and 95% confidence are shown for the number of patients who were categorically classified with a native T1≥1072ms and an ECV≥35% based on the absence or presence of ECG parameters. ECG: Electrocardiogram, ECV: Extracellular Volume, fQRS: Fragmented QRS.
