# Supplementary material for: Association of ECG parameters with late gadolinium enhancement and outcome in patients with clinical suspicion of acute or subacute myocarditis referred for CMR imaging
Source: PLoS One. 2020 Jan 10;15(1):e0227134. doi: 10.1371/journal.pone.0227134 (PMC6953836; doi:10.1371/journal.pone.0227134)
Supplement: S2 Table — Mean±SD or n (%) are shown for ventricular volumes based on the absence or presence of Late Gadolinium Enhancement (LGE). (PDF) [file pone.0227134.s003.pdf]

S2 Table. Supplemental volumetric findings by LGE presentation

	LGE Absent (n=312)	LGE Present (n=275)	p-value
LVEF (%)	52±14	44±16	0.001*
$LVEDV_i$ (ml/m^2)	93±29	106±39	<0.001*
Dilated $LVEDV_i$<br>• >95 ml/m^2 (Females <60years)<br>• >86 ml/m^2 (Females ≥60years)<br>• >100 ml/m^2 (Males <60years)<br>• >94 ml/m^2 (Males ≥60years)	107 (34)	126 (46)	0.016*
Dilated $LVEDV_i$ and LVEF<50%	61 (22)	104 (41)	<0.001*
$LVESV_i$ (ml/m^2)	47±29	64±41	<0.001*
CI (ml/min/m^2)	3165±803	3102±830	0.881
LV $Mass_i$ (g/m^2)	57±15	66±18	0.013*
RVEF (%)	51±8	46±13	<0.001*

Mean±SD or n (%) are shown for ventricular volumes based on the absence or presence of Late Gadolinium Enhancement (LGE).
