# Supplementary material for: Association of ECG parameters with late gadolinium enhancement and outcome in patients with clinical suspicion of acute or subacute myocarditis referred for CMR imaging
Source: PLoS One. 2020 Jan 10;15(1):e0227134. doi: 10.1371/journal.pone.0227134 (PMC6953836; doi:10.1371/journal.pone.0227134)
Supplement: S3 Table — Mean±SD, median [interquartile range] or n (%) are shown for the ECG and CMR findings, along with the univariable hazard ratio and 95% confidence intervals for the association with MACE for patients with acute presentation of symptoms (65 events) vs patients with sub-acute presentation (29 events). (PDF) [file pone.0227134.s004.pdf]

S3 Table. Findings in acute and sub-acute symptom presentation

	Demographics			Univariate Hazard Ratio			
	Acute (<2 weeks)	Sub-Acute (2-4 weeks)	p	Acute (<2 weeks)		Sub-Acute (2-4 weeks)	
**ECG**							
Abnormal ECG	276 (85)	181 (69)	<0.001*	2.20 (0.88-5.48)	0.090	1.72 (0.70-4.24)	0.235
QRS-T Angle (°)	37 [18-90]	35 [16-72]	0.224	1.01 (1.01-1.01)	<0.001*	1.01 (1.00-1.02)	0.005*
Wide QRS-T Angle (≥90°)	80 (25)	52 (20)	0.165	2.34 (1.42-3.87)	0.001*	2.40 (1.13-5.09)	0.022*
Low Voltage	31 (9)	20 (8)	0.462	1.96 (1.00-3.85)	0.051	1.59 (0.55-4.57)	0.394
fQRS	86 (27)	46 (18)	0.009*	0.47 (0.25-0.90)	0.023*	1.72 (0.76-3.91)	0.196
Notched R	56 (18)	30 (12)	0.046*	0.49 (0.22-1.07)	0.074	1.21 (0.42-3.48)	0.730
Notched S	46 (15)	14 (5)	<0.001*	0.64 (0.29-1.40)	0.261	1.66 (0.60-5.52)	0.408
rSr	27 (9)	11 (4)	0.043*	0.29 (0.07-1.20)	0.088	3.57 (1.24-10.3)	0.019*
RSR’	13 (4)	9 (4)	0.828	0.42 (0.06-3.00)	0.384	2.58 (0.61-10.9)	0.198
Fragmented	14 (5)	7 (3)	0.372	0.31 (0.04-2.20)	0.238	1.42 (0.19-10.5)	0.734
Q-wave	48 (15)	29 (11)	0.179	1.15 (0.60-2.20)	0.673	1.75 (0.69-4.61)	0.254
T-wave	111 (35)	64 (24)	0.008*	1.19 (0.72-1.97)	0.400	1.29 (0.59-2.79)	0.522
ST Elevation	24 (8)	8 (3)	0.027*	0.30 (0.07-1.22)	0.902	1.11 (0.15-8.20)	0.918
ST Depression	13 (4)	13 (5)	0.688	0.05 (0.00-5.79)	0.211	2.09 (0.63-6.98)	0.230
PR Depression	8 (3)	2 (1)	0.123	-	-	-	-
PR Duration (ms)	160±28	163±43	0.249	1.00 (0.99-1.01)	0.946	1.00 (1.00-1.01)	0.403
PR Duration (≥200ms)	21 (7)	22 (9)	0.424	1.05 (0.38-2.89)	0.932	2.09 (0.71-6.11)	0.180
QRS Duration (ms)	99±23	102±24	0.098	1.00 (0.99-1.01)	0.611	1.01 (0.99-1.02)	0.411
QRS Duration (≥120ms)	46 (14)	53 (20)	0.076	0.86 (0.41-1.80)	0.682	1.12 (0.46-2.76)	0.804
QTc Duration (ms)	447±44	441±37	0.075	1.01 (1.01-.1.02)	<0.001*	1.00 (1.00-1.01)	0.375
QTc Duration (≥470 female, ≥450 male)	121 (38)	76 (29)	0.028*	2.47 (1.51-4.04)	<0.001*	1.35 (0.63-32.91)	0.441
Left Bundle Branch Block	29 (9)	26 (10)	0.776	0.80 (0.32-1.98)	0.623	0.70 (0.17-2.93)	0.621
Right Bundle Branch Block	24 (7)	18 (7)	0.872	0.50 (0.16-1.59)	0.240	3.28 (1.24-8.63)	0.016*
**Tissue Characterisation**							
LGE Presence	162 (50)	113 (43)	0.081	1.79 (1.98-2.95)	0.024*	2.97 (1.35-6.52)	0.007*
T2w	70 (31)	47 (24)	0.126	2.34 (1.30-4.24)	0.005*	0.96 (0.34-2.67)	0.933
T1 (≥1072ms)	30 (38)	35 (41)	0.750	0.76 (0.24-2.40)	0.638	1.27 (0.30-5.39)	0.749
ECV (≥35%)	22 (31)	23 (28)	0.722	2.68 (0.86-8.42)	0.091	4.82 (1.15-20.2)	0.031*
**Characteristics**							
LVEF (%)	47±15	49±15	0.310	0.96 (0.94-0.97)	<0.001*	0.96 (0.94-0.98)	0.001*
$LVEDV_i$ (ml/m^2)	101±38	97±29	0.170	1.01 (1.00-1.01)	0.018	1.01 (1.00-1.03)	0.040*
$LVESV_i$ (ml/m^2)	57±39	52±30	0.082	1.01 (1.01-1.02)	<0.001*	1.02 (1.01-1.03)	0.003*
CI (ml/min/m^2)	3201±840	3056±779	0.042*	0.99 (0.99-1.00)	0.003*	1.00 (1.00-1.00)	0.305
LV $Mass_i$ (g/m^2)	63±18	59±16	0.003*	1.00 (0.99-1.02)	0.683	1.02 (1.00-1.05)	0.036*
RVEF (%)	48±12	49±10	0.192	0.95 (0.93-0.97)	<0.001*	0.94 (0.91-0.98)	0.002*
Pericardial Effusion	104 (32)	53 (20)	0.001*	2.16 (1.33-3.53)	0.002*	1.68 (0.76-3.69)	0.197
Pleural Effusion	58 (18)	20 (8)	<0.001*	3.85 (2.31-6.44)	<0.001*	2.74 (1.04-7.21)	0.041*
Age (years)	46±16	51±15	<0.001*	1.03 (1.01-1.05)	<0.001*	1.02 (1.00-1.05)	0.119
Sex (female)	125 (39)	117 (44)	0.178	1.75 (1.08-2.85)	0.024*	1.20 (0.58-2.50)	0.618
BMI (kg/m^2)	27.9±6.2	27.4±5.9	0.327	1.06 (1.02-1.10)	0.002*	1.04 (0.98-1.09)	0.193

**Peak Blood Markers**‡							
Creatinine Kinase (U/l)	199 [85-572]	73 [47-102]	0.006*	1.00 (1.00-1.00)	0.539	1.00 (1.00-1.01)	0.021*
C-reactive protein (mg/dl)	16 [4-86]	9 [2-42]	0.005*	1.00 (0.99-1.01)	0.903	0.99 (0.91-1.09)	0.866
NT-proBNP (ng/ml)	1.51 [0.33-5.47]	1.40 [0.15-5.20]	0.884	1.11 (1.06-1.17)	<0.001*	1.15 (1.05-1.26)	0.003*
Troponin (ng/ml)	0.17 [0.01-0.60]	0 [0-0]	<0.001*	1.08 (0.89-1.30)	0.431	-	-
White blood cell count (10^9/l)	9.4 [6.9-13.0]	7.3 [6.0-9.0]	<0.001*	1.01 (0.97-1.06)	0.547	0.95 (0.78-1.16)	0.597

Mean±SD, median [interquartile range] or n (%) are shown for the ECG and CMR findings with a p-value comparing findings between groups, along with the univariable hazard ratio, 95% confidence intervals and p-value for the association with MACE for patients with acute presentation of symptoms (65 events) vs patients with sub-acute presentation (29 events).
